# Testicular Regression Syndrome: Two Case Studies

**DOI:** 10.7759/cureus.34771

**Published:** 2023-02-08

**Authors:** Lamiaa Elazizi, Zineb Elazime, Fatima-Zahra Lahmamssi, Houda Salhi, Hanan Elouahabi

**Affiliations:** 1 Department of Endocrinology, Diabetology, Metabolic Diseases and Nutrition, Hassan II University Hospital Center, Fez, MAR

**Keywords:** laparoscopy, karyotype, amh, testicular agenesis, testicular regression

## Abstract

Testicular agenesis, also called testicular regression syndrome (TRS), is a rare disease. It is defined by the complete absence of testicular tissue associated with a 46,XY karyotype. The phenotype is variable depending on when gonadal regression occurs in utero. Several etiologies have been identified. Here, we report two cases of TRS with an initial diagnosis of cryptorchidism and bilateral impalpable testes. The hormonal assessment showed an undetectable anti-Müllerian hormone (AMH) level and high gonadotropins. Also, radiological exploration did not show the testicles in a normal position, which was confirmed by a negative laparoscopy, establishing the diagnosis of TRS. Androgen replacement therapy along with psychological support to the patient is recommended is such cases.

## Introduction

Bilateral testicular agenesis or bilateral congenital anorchia (BCA) is defined as the absence of partial or complete testicular tissue in the presence of a 46,XY karyotype, and is part of testicular regression syndrome (TRS) [1,2]. Its incidence is estimated to be 0.5-1 in 20,000 men [3,4]. Diagnosis is based on clinical findings, endocrine investigations, cytogenetic analysis, and sometimes surgical exploration. Unfortunately, androgen replacement therapy and prosthetic implantation are the only available therapies for these patients. Herein, we report two cases of bilateral testicular regression syndrome.

## Case presentation

Case 1

A 24-year-old male with a history of treated pleural tuberculosis was followed up from the age of five years for a suspicion of cryptorchidism discovered through the observation of a micropenis with non-palpable testes during a physical examination. The patient was not seen again until he was 15 years old, when he was examined due to delayed puberty. The patient had been treated for micropenis (three monthly testosterone enanthate IM injections at a dose of 100 mg/m²). The clinical examination found the patient in a good general condition, with depressed mood, without dysmorphia, and without macroscelia. His weight was 46.5 kg, with a body mass index of 16.08 kg/m², and height was 1.70 m. The penile length was 7.5 cm (-1.5 SD). Pubic hair was classified as Tanner stage IV, and axillary hair was present. Gynecomastia or spontaneous or induced galactorrhea was not present. Absence of palpable testes in both the intrascrotal and inguinal regions was found.

The karyotype was normal (46,XY) and the SRY gene was normal. His hormonal assessment showed objectively high gonadotropin levels with follicle stimulating hormone (FSH) at 73.9 mIU/mL (normal value, or NV, 1.27-19.26), luteinizing hormone (LH) at 15.37 mIU/mL (NV, 1-7), and an undetectable testosterone level, leading to the conclusion of hypergonadotropic hypogonadism. A human chorionic gonadotropin (HCG) test came out negative, and the anti-Müllerian hormone (AMH) level was found to be very low.

**Figure 1:**
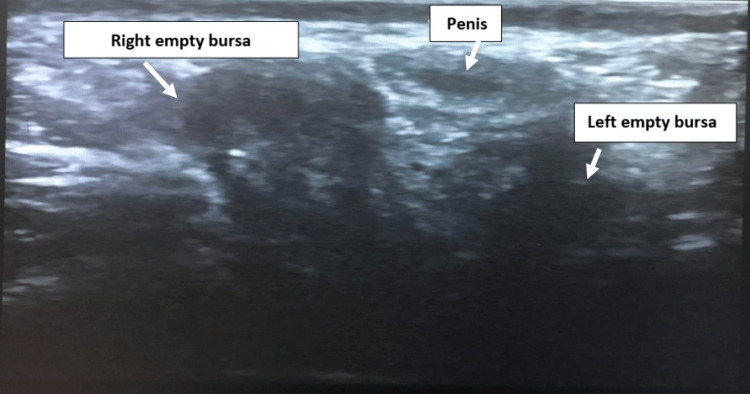
A transverse ultrasound image, in a ventral view through penis and bursae (arrows)

Abdominopelvic and scrotal ultrasounds did not show the testicles in a normal position, which was confirmed by a negative laparoscopy (Figure 1). This confirmed the diagnosis of TRS. Regarding the therapeutic procedure, hormone replacement therapy was initiated (250 mg of testosterone enanthate IM every three weeks) and psychological support was offered before sending him for scrotoplasty.

Case 2

A 36-year-old male had consulted with the urology department (Hassan II University Hospital) at the age of 17 for suspicion of cryptorchidism and was then admitted to our endocrinology department for additional treatment. The clinical examination found the patient in a good general condition, with depressed mood and without dysmorphism. His weight was 81 kg, height 160 cm and body mass index 31.50 kg/m². His facial hair was well-developed. The examination also showed pubic hair at stage V and a 9-cm penis (-1 SD) with a small empty, pleated scrotal bursa.

The patient’s hormonal assessment revealed high gonadotropin levels with FSH at 54.1 mIU/mL (NV, 1.27-19.26) and LH at 23.9 mIU/mL (NV, 1-7), and his testosterone levels were undetectable, leading to the conclusion of hypogonadotropic hypogonadism. Cytogenetic examination revealed a 46,XY karyotype. Karyotyping confirmed the patient to be a 46,XY male with no evidence of mosaicism in the blood cells. PCR amplification of the SRY gene indicated that the patient's SRY gene was normal. The AMH assay showed a level of <0.01 ng/mL (3.1-5.3), and the HCG test was not performed.

The abdominopelvic computed tomography did not reveal the presence of intra-abdominal testicles or any other detectable anomaly. A laparoscopy was subsequently performed and returned negative. In view of these tests, the diagnosis was determined to be that of testicular regression syndrome. Psychological care was first indicated for our patient, and he was then sent for plastic surgery to benefit from a bilateral mastectomy and testicular prosthesis. To address the patient’s hormonal levels, he was put on hormone replacement therapy with testosterone enanthate.

## Discussion

The true prevalence of TRS remains unknown. It is estimated to affect 0.5-1 in 20,000 male births and occurs in 1 out of 177 cases of cryptorchidism [3,4]. Testicular agenesis is a congenital malformation characterised by the absence of testicles due to abnormalities at the start of embryogenesis [3]. Indeed, the foetal testicle must be present during the first 12 weeks of pregnancy for the normal development of male genitalia [4]. Thus, the presence of a normal male phenotype suggests that gonadal regression occurred late in the foetal life, beyond the 12th-14th gestational weeks. Furthermore, rudimentary testicular syndrome develops between the 14th and 20th weeks of pregnancy while anorchia develops after 20 weeks [5]. This is characterised by the internal and external differentiation of the genitalia without the presence of gonadal tissue. This was the case with the two patients in this case study. In both situations, the foetal testicle was functional, ensuring fairly sufficient production of androgen for normal differentiation of the male genital tract and external genitalia through the action of AMH and testosterone. On the other hand, early testicular regression before eight weeks of pregnancy would be due to a female phenotype, and between 8 and 12 weeks would be due to intermediate phenotypes characterised by sexual ambiguity. These forms present the clinician with the problem of sex assignment.

Although the aetiology of testicular agenesis is not fully understood, several hypotheses have been invoked to explain this phenomenon. Some have thought that testicular atrophy results from vascular thrombosis or from testicular torsion in the foetal or perinatal period given the demonstration of macrophages loaded with hemosiderin on the surgical specimens [6]. Furthermore, a combination of a micropenis and an anomaly in the development of the sexual ducts is often present, suggesting the prior presence of an intrinsic alteration of the testicular tissue before its regression. Finally, the description of similar cases in consanguineous families and the association with other congenital malformations suggest a genetic aetiology with autosomal recessive transmission or environmental factors [7]. Indeed, heterozygous mutations of testicular differentiation genes such as WT1, SOX9 and DMRT1 have been described in cases of testicular agenesis in humans. Other genes involved in the development or descent of the gonads (SRY, INSL3 or LGR8) have been suggested, but, to our knowledge, no mutations at these levels have been documented to date [8,9].

Our observations show that the growth and development of children with testicular agenesis before puberty are normal. Timed androgen replacement therapy leads to a pubertal growth spurt with normal secondary sex characteristics and normal bone maturation. A delay in treatment initiation after normal puberty bone age leads to the development of eunuchoid body proportions.

Biologically, the AMH level can be used as a marker for the presence of testicular tissue. Extremely low or undetectable AMH levels with an absence of the plasma testosterone response to stimulation by HCG can indicate TRS. Misra et al. reported a higher specificity and positive predictive value of AMH compared to the HCG test for diagnosis [10]. The levels of gonadotropins, FSH and LH are found to be high. The combination of undetectable AMH levels and high gonadotropins during puberty is very likely in the diagnosis of TRS. However, an HCG stimulation test is necessary in prepubertal children, whose gonadotropin levels are not high, to eliminate the rare cases of persistence of Müller's ducts, or dosage errors. In the first patient, we noted perfect concordance between the AMH results and the HCG stimulation test. In the second patient, for whom the stimulation test was not performed, laparoscopic exploration was indicated, which subsequently confirmed the diagnosis. Ultrasound and magnetic resonance imaging may sometimes fail to show testicular tissue [11,12]. Diagnostic confirmation can only be provided by laparoscopic exploration, the use of which remains controversial [2,8,13,14]. When it is carried out, it can confirm the absence of gonads, as was the case for our patients, or the presence of rudimentary testicles. It can also confirm the existence of a normal arrangement of the spermatic pedicle and the vas deferens, testifying to normal migration of the testicle that was destroyed late in the foetal life [13].

Given the negligible risk of malignant degeneration of the testicular tissue, which is only found in 10% of explorations, and without germ cells on the surgical specimens, some authors suggest that laparoscopic exploration is not systematic [15]. However, other authors reported the presence of germ cells in 11% of cases and seminiferous tubules in 0 to 40%, with viability in 0 to 16% of cases. This raises fears of a potential risk of malignant degeneration, thus justifying laparoscopic exploration. We also believe that laparoscopy should always be indicated, which was carried out in our first patient, to be able to conclude the status of the gonads definitively, thus obviating other investigations.

Patient management requires a multidisciplinary team approach. Androgen replacement therapy is recommended at the time of puberty. The presence of a micropenis, as in our patients, requires androgen therapy to be initiated as soon as possible to avoid further burdening their psychological experience in adolescence and adulthood. Indeed, psychological evaluation and advice from the parents at diagnosis are necessary. Implantation of a testicular prosthesis can be envisaged for psycho-social and aesthetic reasons but should be carried out before the start of replacement treatment. This technique carries possible complications, such as inflammation or perforation of the skin. Finally, psychological support should be offered to patients and their families.

## Conclusions

Testicular regression syndrome is a rare condition that is defined by the complete absence of testicular tissue in a patient with a normal male karyotype. The phenotype is variable depending on when gonadal regression occurs in utero. Its aetiology is still debated but its familial occurrence is an argument to suggest a genetic aetiology. The diagnostic approach of this entity requires paraclinical investigations or even laparoscopy. The therapeutic possibilities are currently limited to androgen replacement therapy that could be combined with the implantation of a prosthesis. It is necessary to initially give psychological support to the parents and then to the patient himself, thus improving the experience of the pathology.
